# Circulating Estradiol Regulates Brain-Derived Estradiol via Actions at GnRH Receptors to Impact Memory in Ovariectomized Rats

**DOI:** 10.1523/ENEURO.0321-16.2016

**Published:** 2016-12-13

**Authors:** Britta S. Nelson, Katelyn L. Black, Jill M. Daniel

**Affiliations:** 1 Neuroscience Program, Tulane University, New Orleans, LA 70118; 2 Brain Institute, Tulane University, New Orleans, LA 70118; 3 Department of Psychology, Tulane University, New Orleans, LA 70118

**Keywords:** estradiol, estrogen, GnRH, hippocampus, memory

## Abstract

Systemic estradiol treatment enhances hippocampus-dependent memory in ovariectomized rats. Although these enhancements are traditionally thought to be due to circulating estradiol, recent data suggest these changes are brought on by hippocampus-derived estradiol, the synthesis of which depends on gonadotropin-releasing hormone (GnRH) activity. The goal of the current work is to test the hypothesis that peripheral estradiol affects hippocampus-dependent memory through brain-derived estradiol regulated via hippocampal GnRH receptor activity. In the first experiment, intracerebroventricular infusion of letrozole, which prevents the synthesis of estradiol, blocked the ability of peripheral estradiol administration in ovariectomized rats to enhance hippocampus-dependent memory in a radial-maze task. In the second experiment, hippocampal infusion of antide, a long-lasting GnRH receptor antagonist, blocked the ability of peripheral estradiol administration in ovariectomized rats to enhance hippocampus-dependent memory. In the third experiment, hippocampal infusion of GnRH enhanced hippocampus-dependent memory, the effects of which were blocked by letrozole infusion. Results indicate that peripheral estradiol-induced enhancement of cognition is mediated by brain-derived estradiol via hippocampal GnRH receptor activity.

## Significance Statement

Results of years of research have demonstrated that circulating estradiol, the main estrogen produced by the ovaries, impacts memory and the hippocampus. More recently, data have revealed that the brain can synthesize its own estradiol, the release of which also impacts the hippocampus. The relationship between the ability of circulating estradiol and brain-derived estradiol to impact the hippocampus and hippocampus-dependent memory is unclear. Here we demonstrate that the impact of circulating estradiol on memory is mediated by brain-derived estradiol through mechanisms involving gonadotropin-releasing hormone (GnRH). These data have significance for understanding mechanisms by which loss of ovarian hormones can impact memory.

## Introduction

17β-Estradiol (estradiol), the primary estrogen synthesized by the ovaries, impacts memory and the hippocampus (Luine, 2014). Specifically, systemic estradiol treatment to ovariectomized rats enhances performance on a variety of tasks designed to measure hippocampus-dependent memory (Daniel and Bohacek, 2010; Boulware et al., 2012). The ability of circulating estradiol to enhance performance on hippocampus-dependent tasks is associated with structural changes in the hippocampus that are hypothesized to correlate with memory formation (Spencer et al., 2008). For example, ovariectomy decreases dendritic spine density in region CA1 in rat hippocampus, and systemic estradiol administration reverses these effects (Gould et al., 1990). Additionally, dendritic spine density fluctuates throughout the estrous cycle of rats, with higher levels of estradiol corresponding to greater hippocampal dendritic spine density (Woolley et al., 1990). Results of these early studies as well as many subsequent ones (Smith et al., 2009) implicate ovarian estrogens as mediators of hippocampal synaptic plasticity.

More recent work has called into question this traditional view, that estrogens derived from the periphery impact hippocampal synapse structure (see Fester and Rune, 2014). All proteins required for estradiol synthesis are located in the hippocampus, including aromatase, the enzyme responsible for the conversion of testosterone to estradiol (Furukawa et al., 1998; Fester et al., 2011; Tabatadze et al., 2014). Whereas application of physiological or supraphysiological levels of estradiol to hippocampal slices had no impact on dendritic spine density (Kretz et al., 2004), inhibition of hippocampal estradiol synthesis via aromatase inhibitors decreased dendritic spine density (Prange-Kiel et al., 2003) and synaptic protein expression (Prange-Kiel et al., 2006), suggesting that hippocampus-derived and not peripherally derived estradiol is critical in maintaining hippocampal synapses.

Data implicating a role for gonadotropin-releasing hormone (GnRH) in hippocampal synaptic plasticity may reconcile conflicting evidence regarding impacts of peripheral versus brain-derived estradiol in the hippocampus. For instance, release of GnRH is regulated by peripheral estradiol (Terasawa and Kenealy, 2012), and the hippocampus expresses high levels of GnRH receptors on cells capable of estradiol synthesis (Fester et al., 2012). GnRH treatment in rat hippocampal slices induces long-lasting enhancement of synaptic transmission in CA1, effects of which are mediated by local estradiol synthesis, as application of an aromatase inhibitor blocks the effect (Wang et al., 2010; Schang et al., 2011). Furthermore, GnRH treatment dose-dependently stimulated estradiol synthesis in the hippocampus, thereby affecting dendritic spine density and levels of the synaptic proteins synaptophysin and spinophilin, effects of which were blocked by inhibiting estradiol synthesis (Fester et al., 2012; Prange-Kiel et al., 2013). Collectively, these results indicate that GnRH regulates hippocampal neuroestradiol synthesis, which in turn affects hippocampal morphology. The subsequent effect of these neural changes on memory has not been examined.

The goal of the current experiments was to test the hypothesis that circulating estradiol impacts hippocampus-dependent memory through regulation of hippocampus-derived estradiol via mechanisms involving GnRH. In an initial experiment, we inhibited brain estradiol synthesis via administration of an aromatase inhibitor to ovariectomized rats that were treated systemically with estradiol to determine whether brain estradiol is necessary for circulating estradiol to enhance memory. Next, we administered a GnRH receptor antagonist to the dorsal hippocampus of ovariectomized estradiol-treated rats to determine whether hippocampus GnRH receptor activation is necessary for circulating estradiol to enhance memory. Finally, we determined whether a relationship exists between effects of GnRH and neuroestradiol on hippocampus-dependent memory. We administered GnRH or GnRH plus an aromatase inhibitor into the dorsal hippocampus of ovariectomized rats to establish, first, whether GnRH receptor activation in the absence of circulating estradiol is sufficient to enhance memory and, second, whether GnRH effects are dependent on neuroestradiol synthesis.

## Materials and Methods

### Subjects and hormone treatments

**Figure 1.**
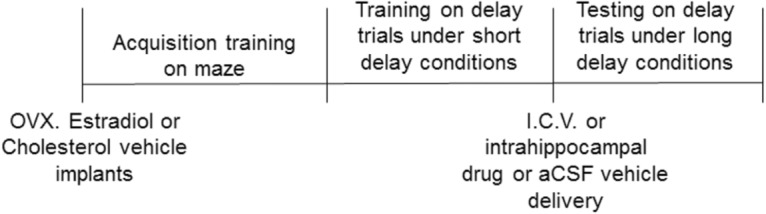
Experimental timeline used in all experiments. OVX, ovariectomy.

Female Long-Evans hooded rats, approximately 2 months of age, were purchased from Harlan Sprague Dawley (Indianapolis, IN). Animal care was in accordance with guidelines set by the *National Institutes of Health Guide for the Care and Use of Laboratory Animals*, and all procedures were approved by the Institutional Animal Care and Use Committee of Tulane University. Rats were housed individually in a temperature-controlled vivarium under a 12-h light/dark cycle (lights on at 7:00 a.m.). One week after arrival, all rats were ovariectomized while under anesthesia induced by injections of ketamine (100 mg/kg i.p., Bristol Laboratories, Syracuse, NY) and xylazine (7 mg/kg i.p., Miles Laboratories, Shawnee, KS). At the time of surgery, animals were implanted with 5 mm Silastic brand capsules (0.058-inch i.d. and 0.077-inch o.d., Dow Corning, Midland, MI) containing either 25% 17β-estradiol (Sigma-Aldrich, St. Louis, MO) diluted with cholesterol or 100% cholesterol vehicle. We have reported previously that estradiol implants of these dimensions maintain blood plasma estradiol levels of 26–47 pg/ml, which fall within the physiological range of cycling female rats (Bohacek and Daniel, 2007, 2010) . See Fig. 1 for experimental timeline.

### Maze training: acquisition

One week after ovariectomy, rats were placed on diets to maintain body weights at 85–90% of presurgery weights and were trained to obtain food rewards (Froot Loops; Kellogg Co., Battle Creek, MI) from the arms of an elevated eight-arm radial maze purchased from Lafayette Instrument Co. (Lafayette, IN). The maze consists of black metal floors and clear acrylic walls with arms (10 cm wide × 70 cm long × 20 cm high) extending out from an octagonal center (33 cm across). The maze is located in the center of a 3 × 5 m room and raised ∼1 m from the floor. Several extramaze cues, including overhead fluorescent lights, desk, chairs, sink, and door, were visible from the maze. To begin a trial, a rat was placed in the center compartment in a pseudorandom orientation and had access to all eight arms. Arm choices were recorded by an observer seated in a fixed location approximately 1 m away from the maze. An arm choice was scored if the rat traversed half the length of an arm. Rats were allowed to choose arms in any order until all arms had been visited or 5 min had elapsed. Errors were reentries into previously visited arms. Each animal received one trial per day across 24 days of acquisition.

### Maze training: short delays

After acquisition training was completed, rats were trained on delay trials under short delay conditions to acclimate them to delay trial procedures that would later be used to test treatment effects. For delay trials, an animal was removed from the maze after the fourth correct arm choice and put in a holding cage for delays of 1 or 30 min, after which it was returned to the maze until the four remaining, still baited arms, were visited or until 5 min had elapsed. Consequently, animals had to remember over an extended period of time which arms had already been entered. Arm choice accuracy was measured by the number of errors of the first eight arm choices. Rats were given one day of habituation to a 1-min delay trial. Subsequently, two trials (one per day) were conducted for each delay beginning with a 1-min delay.

### Drug treatments

After completing short-delay trials on the radial maze, drug treatments began. Drugs or vehicle were delivered to the lateral ventricle or the dorsal hippocampus as described below. Rats were anesthetized with ketamine and xylazine and placed in a stereotaxic frame.

#### Experiment 1: Letrozole

A hole was drilled in the skull, and cannulae (Brain Infusion Kits, Alzet, Cupertino, CA) were lowered through the hole to the appropriate depth (to the left lateral ventricle located –0.3 mm AP, –1.2 mm ML, and –4.5 mm DV) and anchored to the skull with screws and dental acrylic. Cannulae were connected to Alzet osmotic minipumps by vinyl tubing that delivered artificial cerebrospinal fluid (aCSF) vehicle (Tocris Bioscience, Ellisville, MO) or letrozole (1.67 μg/μl; Bachem, Torrance, CA), an aromatase inhibitor, dissolved in dimethylsulfoxide and diluted in vehicle at a rate of 0.5 μl/h. All pumps were implanted s.c. in the nape of the neck, and cannulae were inserted after the pumps began pumping. Cholesterol-treated rats received osmotic minipumps containing letrozole (CH letrozole, *n* = 10), or vehicle aCSF (CH aCSF, *n* = 14). Estradiol-treated rats received osmotic minipumps containing letrozole (E letrozole, *n* = 12) or vehicle aCSF (E + aCSF, *n* = 13). To facilitate procedures, surgeries, and subsequent behavior testing were staggered across four cohorts. All groups were represented in all cohorts.

#### Experiment 2: Antide

Holes were drilled in the skull, and a 10-µl Hamilton syringe was lowered through each hole to the appropriate depth to the left and right dorsal hippocampus (–3.3 mm AP, ±1.5 mm ML, and –2.0 mm DV). The long-lasting GnRH receptor antagonist, antide, diluted in aCSF (1 µg/µl; Sigma-Aldrich) was infused bilaterally via syringe at a rate of 1 µl/min over a period of 2.5 min. Syringes remained in place for an additional minute to ensure diffusion of the drug. The dose of antide was based on a report indicating a single hypothalamic infusion of antide blocked estrous cycles in rats, an effect that persisted from 11 days to >4 months, indicating its long-term effectiveness (Weesner and Pfaff, 1994). Half of the cholesterol-treated rats received antide infusions (CH Antide, *n* =10), and half received vehicle aCSF (CH aCSF, *n* = 10). Half of the estradiol-treated rats received antide infusions (E Antide, *n* = 10) and half received vehicle aCSF (E aCSF, *n* = 10). To facilitate procedures, surgeries, and subsequent behavior testing were staggered across two cohorts. All groups were represented in both cohorts. One rat (CH Antide) died prior to data collection due to surgical complications.

To confirm that potential effects of intrahippocampal antide infusions were due to impacts in the hippocampus and generalized effects via spread of the drug to ventricles, we infused antide following identical procedures as described above to the hippocampus of two gonadally intact female rats. Analyses of daily vaginal smears collected by lavage beginning 1 week after the antide infusions revealed that both rats continued to display regular 4-d estrous cycles. These results provided evidence that intrahippocampal administered antide was not reaching the hypothalamus, where it would disrupt the estrous cycle of the rat, suggesting that our regimen of antide administration did not result in spread of drug to the ventricles.

#### Experiment 3: GnRH and GnRH + letrozole

Holes were drilled in the skull, and cannulae (Brain Infusion Kits, Alzet) were lowered through the holes to the appropriate depth (to the left and right dorsal hippocampi, –3.3 mm AP, ±1.5 mm ML, and –2.0 mm DV) and anchored to the skull with screws and dental acrylic. Cannulae were connected to Alzet osmotic minipumps by vinyl tubing that delivered artificial aCSF vehicle (*n* = 8), GnRH (16.6 ng/h; Sigma-Aldrich; *n* = 9) or GnRH + letrozole (31.5 ng/h) diluted in vehicle delivered at a rate of 0.25 μl/h (*n* = 9). All pumps were implanted s.c. in the nape of the neck, and cannulae were inserted after the pumps began pumping. To facilitate procedures, surgeries, behavior testing, and sacrifice were staggered across two cohorts. All groups were represented in both cohorts.

### Behavioral testing: long-delay trials

One week after initiation of drug treatments, behavioral testing began. Behavioral testing consisted of long-delay trials during which delays of 2 and 4 h were imposed between the fourth and fifth arm choices. Two trials were conducted for each delay using procedures identical to those used for the short-delay trials previously described. We chose to assess effects of drug treatments only under the two long-delay conditions and not during shorter delays because of time constraints related to use of brain infusion kits and because previous work by us (Daniel et al., 2005) and others (Bimonte and Denenberg, 1999) indicated that effects of estradiol on memory are most apparent under conditions of high memory demand.

### Hormone treatment efficacy

At the time the rats were killed, uteri were extracted and 1-cm-long sections of the right uterine horns (cut at the base) were weighed to verify efficacy of hormone treatment. Additionally, proper removal of the ovaries and integrity of the implanted capsules were assessed.

### Statistical analyses

Arm-choice accuracy data from short-delay trials were analyzed by two-way ANOVA (hormone × delay) with repeated measures on delay for Experiments 1 and 2 and one-way repeated measures ANOVA (delay) for Experiment 3. Arm-choice accuracy data from long-delay trials in Experiments 1 and 2 were analyzed by three-way ANOVA (hormone × drug × delay) with repeated measures on delay. *Post hoc* tests [Fisher’s least significant difference (LSD), *p* < 0.05] were used to probe group differences following significant interactions of hormone × drug. Arm-choice accuracy data from long-delay trials in Experiment 3 were analyzed by two-way ANOVA (drug × delay) with repeated measures on delay. Fisher’s LSD *post hoc* tests were used to probe group differences following a main effect of drug. A one-way ANOVA, with hormone group as the factor, was used to test for differences in uterine weight in Experiments 1 and 2.

## Results

### Experiment 1

The goal of the first experiment was to test the hypothesis that the ability of circulating estradiol to impact hippocampus-dependent memory was dependent on neuroestradiol. Thus, we determined whether central delivery of the aromatase inhibitor letrozole would block effects of peripherally administered estradiol to ovariectomized rats on radial-maze performance. Rats were ovariectomized and received subcutaneous estradiol or cholesterol vehicle capsule implants. After training on short-delay trials in the radial-maze, rats received chronic intracerebroventricular (i.c.v.) delivery of vehicle or letrozole via cannulae connected to osmotic minipumps. Rats then were tested on long-delay trials on the radial-arm maze.

#### Short-delay trials (before drug treatment)

**Figure 2.**
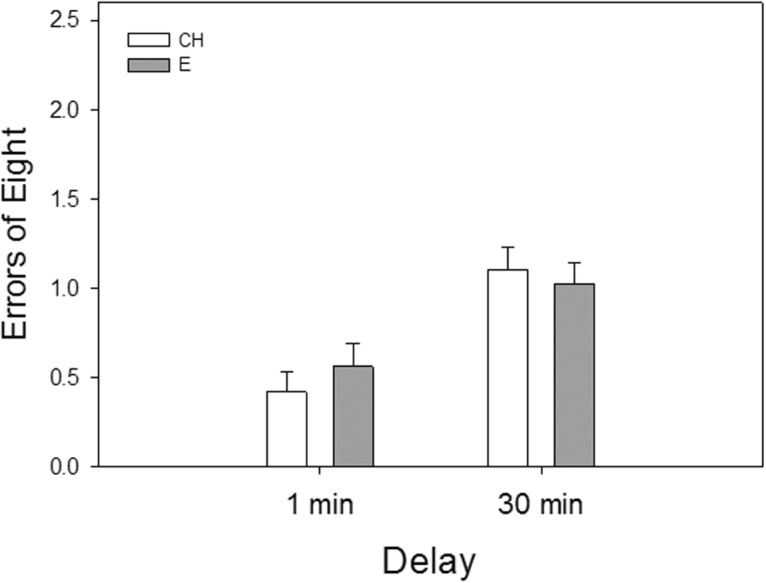
Working memory performance of ovariectomized estradiol-treated (E) and cholesterol vehicle-treated (CH) ovariectomized rats in a radial-arm maze during training on short-delay trials in Experiment 1. Delays were imposed between the fourth and fifth arm choices. Mean number of errors of the first eight arm choices (±SEM) at each delay. *p < 0.05 vs. 1-min delay.

Analyses of arm-choice accuracy data from training on the two short-delay trials indicated a main effect of delay (*F*_(1,47)_ = 33.88, *p* = 0.0001) indicating an increased numbers of errors on the longer delay (Fig. 2). There was no effect of hormone or an interactive effect of hormone by delay, indicating that estradiol treatment did not affect performance during short-delay trials.

#### Long-delay trails (during drug treatment)

**Figure 3.**
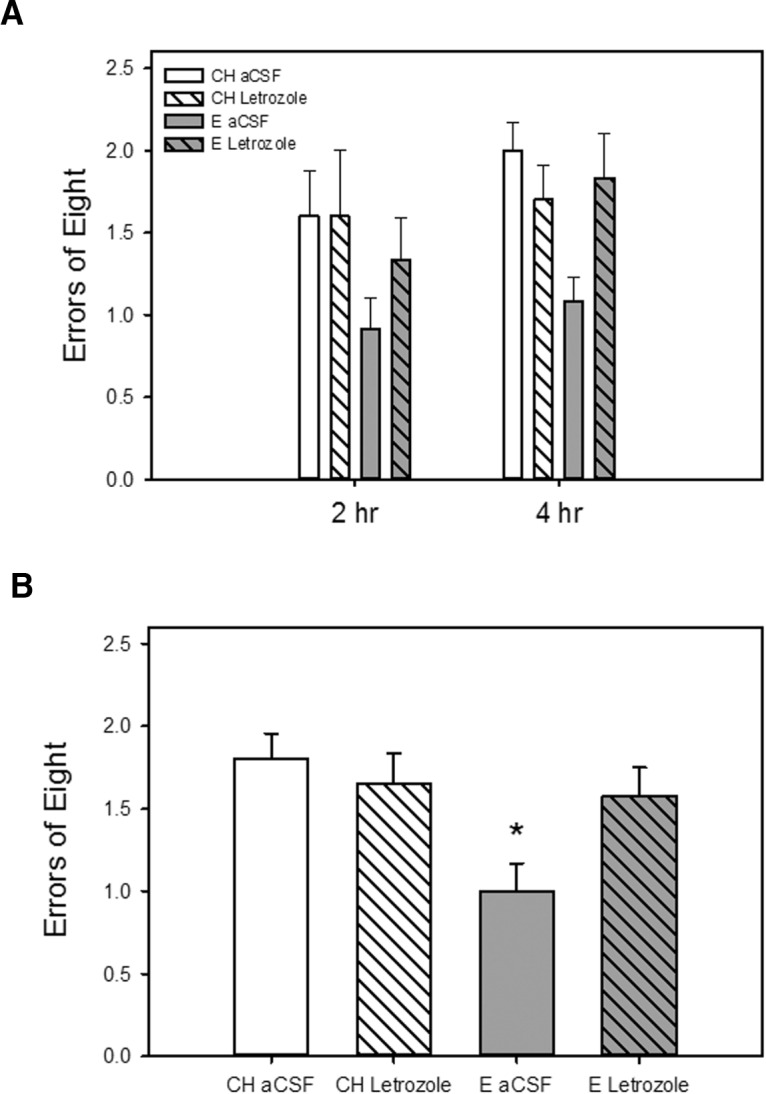
Effects of estradiol treatment and inhibition of brain estradiol synthesis on a hippocampus-dependent spatial memory task. Rats were ovariectomized and implanted with capsules containing estradiol (E) or cholesterol vehicle (CH). Chronic intracerebroventricular delivery of the aromatase inhibitor letrozole or aCSF vehicle was initiated 1 week before and maintained throughout testing. ***A***, Working memory performance in a radial-arm maze with delays imposed between the fourth and fifth arm choices. Mean number of errors of the first eight arm choices (±SEM) at each delay. ***B***, Mean number of errors of the first eight arm choices (±SEM) averaged across both delays. *p < 0.05 vs. all other groups.

As illustrated in Fig. 3, chronic i.c.v. administration of letrozole blocked an estradiol-induced enhancement in memory as assessed on a radial-arm maze during testing across long-delay trials when delays of 2 and 4 h were imposed between the fourth and fifth arm choices. There was a significant main effect of hormone (*F*_(1,45)_ = 5.94, *p* = 0.019) on maze performance as measured by number of errors. There was also a significant interactive effect of hormone × drug (*F*_(1,45)_= 4.22, *p* = 0.046), indicating that the effect of estradiol treatment differed depending on whether letrozole was administered. *Post hoc* analyses revealed that rats that received estradiol treatment and control i.c.v. aCSF infusion (E aCSF) had significantly better arm choice accuracy (fewer errors) than rats that received cholesterol control treatment and aCSF or letrozole infusion (CH aCSF, CH Letrozole) as well as rats that received estradiol treatment and letrozole infusion (E Letrozole). There were no main effects of drug or delay.

#### Hormone treatment efficacy

There was a significant difference between groups in uterine weight (*F*_(1,47)_ =504.82, *p* < 0.001), indicating that hormone treatments were effective. Estradiol-treated rats had larger uteri (mean ± SEM; 68.9 ± 3.04 mg) than cholesterol-treated rats (18.7 ± 3.27 mg).

### Experiment 2

Results of our first experiment revealed that synthesis of neuroestradiol was a necessary component in the ability of circulating estradiol to enhance memory. We hypothesized that GnRH, which is regulated by circulating estradiol and can impact levels of neuroestradiol (Terasawa and Kenealy, 2012), is involved in the enhancement. Thus, to explore the importance of GnRH in the effects of circulating estradiol on memory, we assessed the ability of antagonizing GnRH receptors in the hippocampus to block the impact of circulating estradiol on memory. Rats were ovariectomized and received subcutaneous estradiol or cholesterol vehicle capsule implants. After training on short-delay trails in the radial maze, rats received a single bilateral infusion of vehicle or antide, a long-lasting GnRH receptor antagonist, to the dorsal hippocampus. Rats then were tested on long-delay trials in the maze.

#### Short-delay trials (before drug treatment)

Analyses of arm-choice accuracy data from training on the two short-delay trials showed a main effect of delay (*F*_(1,37)_ = 8.45, *p* = 0.006), indicating an increased numbers of errors on the longer delay. There was no effect of hormone or an interactive effect of hormone by delay, indicating that estradiol treatment did not affect performance during short-delay trials.

#### Long-delay trails (after drug treatment)

**Figure 4.**
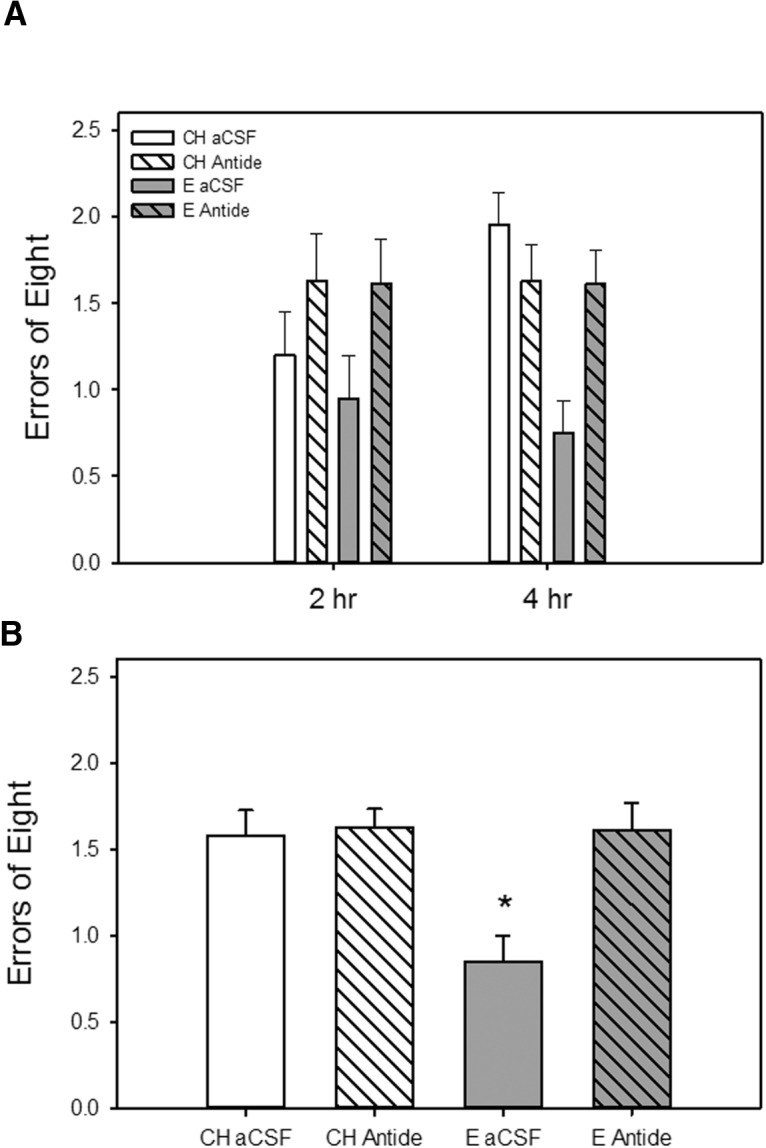
Effects of estradiol treatment and antagonism of hippocampal GnRH receptors on a hippocampus-dependent spatial memory task. Rats were ovariectomized and implanted with capsules containing estradiol (E) or cholesterol vehicle (CH). A single bilateral infusion of the long-lasting GnRH receptor antagonist antide or aCSF vehicle was delivered to the hippocampus 1 week before the initiation of testing. ***A***, Working memory performance in a radial-arm maze with delays imposed between the fourth and fifth arm choices. Mean number of errors of the first eight arm choices (±SEM) at each delay. ***B***, Mean number of errors of the first eight arm choices (±SEM) averaged across both delays. *p < 0.05 vs. all other groups.

As illustrated in Fig. 4, intrahippocampal infusion of antide, a long-lasting GnRH receptor antagonist, blocked an estradiol-induced enhancement in memory as assessed on a radial-arm maze during testing across long-delay trials. Data from two rats (CH antide, E antide) were excluded from the long-delay data analyses because they did not complete either testing trial on the 4-h delay. There were significant main effects of hormone (*F*_(1,33)_ = 5.47, *p* = 0.025), and drug (*F*_(1,33)_ = 6.59, *p* = 0.015) on maze performance as measured by number of errors. There was also a significant interactive effect of hormone × drug (*F*_(1,33)_= 5.07, *p* = 0.031), indicating that the effect of estradiol treatment differed depending on whether antide was administered. *Post hoc* analyses revealed that the rats that received estradiol treatment and control aCSF infusion (E aCSF) had significantly better arm choice accuracy (fewer errors) than rats that received cholesterol control treatment and aCSF or antide infusion (CH aCSF, CH antide) as well as rats that received estradiol treatment and antide infusion (E antide). There were no effects of delay.

#### Hormone treatment efficacy

There was a significant difference between groups in uterine weight (*F*_(1,35)_ =310.94, *p* < 0.001), indicating that hormone treatments were effective. Estradiol-treated rats had larger uteri (mean ± SEM; 69.8 ± 1.12 mg) than cholesterol-treated rats (12.8 ± 2.97 mg).

### Experiment 3

Results of Experiments 1 and 2 demonstrated that neuroestradiol synthesis and action at hippocampal GnRH receptors are each necessary for circulating estradiol to impact memory. In Experiment 3, we investigated whether there was an association between these two effects. Specifically, we determined whether GnRH delivered to the hippocampus in the absence of circulating estradiol was sufficient to enhance memory and whether effects of GnRH on memory were dependent on neuroestradiol synthesis. Rats were ovariectomized, and all received subcutaneous cholesterol vehicle capsule implants. After training on short-delay trials in the radial-arm maze, rats received chronic intrahippocampal delivery of vehicle, GnRH, or GnRH + letrozole via cannulae connected to osmotic minipumps. Rats then were tested on long-delay trials in the maze.

#### Short-delay trials (before drug treatment)

Analyses of arm-choice accuracy data from training on the two short-delay trials revealed no effect of delay indicating that the increase in delay did not significantly impact performance in ovariectomized cholesterol vehicle-treated rats.

#### Long-delay trails (during drug treatment)

**Figure 5.**
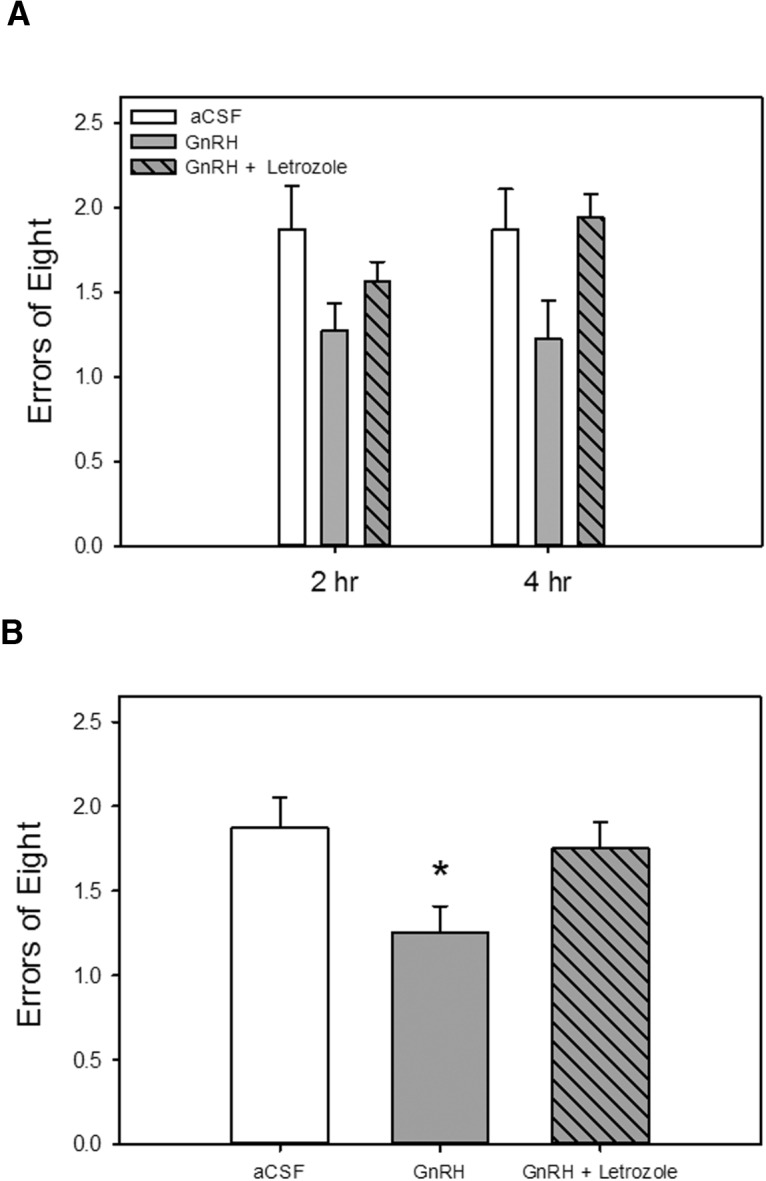
Effects of intrahippocampal delivery of GnRH alone or with an aromatase inhibitor on performance on a hippocampus-dependent spatial memory task. Rats were ovariectomized, and all were implanted with capsules containing cholesterol vehicle. Chronic bilateral intrahippocampal delivery of GnRH, GnRH + the aromatase inhibitor letrozole, or aCSF vehicle was initiated 1 week before and maintained throughout testing. ***A***, Memory performance in a radial-arm maze with delays imposed between the fourth and fifth arm choices. Mean number of errors of the first eight arm choices (±SEM) at each delay. ***B***, Mean number of errors of the first eight arm choices (±SEM) averaged across both delays. *p < 0.05 vs. other groups.

As illustrated in Fig. 5, results indicate that hippocampal GnRH exerts cognitive benefits that are dependent on hippocampal estradiol synthesis. Analyses revealed a significant main effect of treatment group (*F*_(1,23)_ = 3.81, *p =* 0.037) on maze performance as measured by number of errors. *Post hoc* analyses revealed that the rats that received GnRH infusion had significantly better arm choice accuracy (fewer errors) than rats that received aCSF control infusion or GnRH + letrozole infusion. There were no effects of delay.

#### Hormone treatment efficacy

Atrophy of uteri of all animals was confirmed at the time of euthanasia.

## Discussion

Results of the present experiments indicate that the positive impact on spatial memory of chronic systemic estradiol treatment in ovariectomized rats is mediated by brain-derived estradiol. Further, the current data provide evidence that the relationship between the effects of circulating estrogens and brain estrogens on memory is mediated by GnRH. Specifically, we showed that blocking brain-derived estradiol synthesis via letrozole, an aromatase inhibitor, blocked the ability of systemic estradiol treatment to ovariectomized rats to enhance performance on a spatial radial-arm maze task. Antagonizing hippocampal GnRH receptors also blocked the estradiol-induced effects on performance, demonstrating that hippocampal GnRH receptor signaling is required for peripheral estradiol treatment to enhance memory. Furthermore, hippocampal GnRH treatment enhanced memory in ovariectomized rats in the absence of peripherally administered estrogens. This enhancement in memory was blocked by letrozole, indicating that GnRH is exerting benefits on memory by impacting neuroestradiol synthesis in the hippocampus. Collectively, our data provide support for the hypothesis that circulating estrogens impact cognition by acting to mediate brain levels of GnRH, which in turn acts at receptors in the hippocampus to facilitate synthesis of neuroestradiol, which then impacts hippocampus-dependent memory.

The current data are consistent with previous work indicating that chronic systemic administration of estradiol to ovariectomized rats enhances spatial memory, an effect that is particularly evident as memory load increases (Bimonte and Denenberg, 1999; Daniel et al., 2005). In Experiments 1 and 2, estradiol compared with vehicle treatment enhanced performance on an eight-arm radial-maze task when long delays (i.e., 2 and 4 h) were imposed between the fourth and fifth arm choices. Results of Experiment 1 revealed that chronic systemic estradiol treatment acts via regulation of brain estradiol to exert its impact on memory, as inhibition of brain estradiol synthesis via central administration of letrozole blocked the effect. Importantly, the negative impact on memory of blocking brain estradiol was specific to rats receiving exogenous administration of estradiol. Letrozole administration had no effect on cognitive performance in ovariectomized controls, suggesting that brain estrogens do not impact hippocampus-dependent memory in the absence of circulating estrogens. In the present study, rats had been ovariectomized ∼2 months before letrozole delivery. It remains to be determined whether neuroestradiol inhibition would impact memory after shorter periods of circulating estradiol deprivation.

The present data indicate that peripheral estradiol administration exerts effects on memory by regulating neuroestradiol synthesis. Supportive of the results are reports that levels of estradiol are present in higher concentrations in the hippocampus than in the periphery (Hojo et al., 2009) and are associated with levels of circulating estrogens (Fester et al., 2012). We hypothesize that the impact of neuroestradiol on memory is due to specific actions in the hippocampus. But because we delivered letrozole to the lateral ventricle, we cannot rule out the possibility that brain-derived estrogens synthesized elsewhere in the brain contributed to our observed effects. For example, neuroestradiol synthesized in the hypothalamus is critical in mediating the effects of peripheral estradiol on GnRH release in female primates (Kenealy et al., 2013), which could potentially reach the hippocampus to exert effects. Nevertheless, our data demonstrate that effects of exogenously administered circulating estradiol on cognition are mediated by brain-derived estrogens.

Our results are in contrast to results recently reported in which acute inhibition of hippocampal synthesis of estrogens via letrozole administration to ovariectomized mice impaired object memory consolidation and had no impact on the ability of i.c.v. administration of estradiol to enhance memory consolidation (Tuscher et al., 2016). In addition to differences in tasks and memory processes assessed, there are differences in treatment paradigms that may have contributed to these discrepant results. We administered letrozole chronically and to the lateral ventricles, whereas Tuscher et al. administered a single infusion to the hippocampus. Additionally, it is likely that the weeks of chronic circulating estradiol treatment in the current work would have a different impact on the brain and on hippocampus-derived estrogens than a single i.c.v. infusion of estradiol used in the previous report.

In the current work, results of Experiment 2 revealed that intrahippocampal delivery of antide, a GnRH receptor antagonist, blocked the ability of systemically administered estradiol to ovariectomized rats to impact performance on a hippocampus-dependent task. In parallel to results of letrozole treatment in Experiment 1, the impact of antide was specific to estradiol-treated females. These results support a specific role for GnRH actions in the hippocampus in the mediation of circulating estradiol-induced effects on memory. The current data support previous work on the effects of GnRH on learning and memory whereby both peripheral and central administration of GnRH to aged male and female mice increased neurogenesis throughout the brain as well as enhanced hippocampus-dependent memory (Zhang et al., 2013).

Circulating estrogens regulate GnRH release through both positive and negative feedback mechanisms. In normally cycling female rats, estradiol exerts negative feedback on GnRH release during the early follicular phase of the estrous cycle (Clarke and Cummins, 1987) by acting at the level of the arcuate nucleus in the hypothalamus, interacting with kisspeptin neurons which synapse directly on GnRH neurons (Smith et al., 2005; Roa et al., 2008). As estradiol levels rise, they act to increase release of GnRH via positive feedback mechanisms that correspond to ovulation. This surge of GnRH is due to indirect actions of estradiol on kisspeptin neurons in the anteroventral periventricular (AVPV) nucleus of the hypothalamus (Roa et al., 2009). In the current experiments, rats were ovariectomized and administered constant estradiol at physiological levels. As such, the feedback mechanisms on GnRH neurons differ from those of normally cycling gonadally intact rodents. In gonadectomized rodents, continuous estradiol treatment exerts both negative and positive feedback on a daily basis rather than over a more extended period across the 4-d estrous cycle (Legan and Karsch, 1975). As such, rats continuously treated with estradiol would likely experience an increased level of GnRH receptor activation compared with normally cycling rodents.

Although our data point to actions of GnRH in the hippocampus, the potential source of GnRH to the hippocampus is unclear. The possibility of direct projections between the hypothalamus and hippocampus is still under debate (Colom et al., 2005; Prange-Kiel et al., 2013), although early work has found GnRH-immunopositive processes to the hippocampus in female rats (Merchenthaler et al., 1984). Additionally, GnRH is present in cerebral spinal fluid, indicating that it can have widespread effects throughout the brain via diffusion through the ventricles (Skinner and Caraty, 2002). In the present studies, GnRH is proposed to impact memory by acting in the hippocampus to regulate neuroestradiol synthesis. A possible mechanism by which GnRH regulates neuroestradiol synthesis is through increasing levels of steroid acute regulatory protein (StAR), which initiates neurosteroid synthesis (Rosati et al., 2011). Further, GnRH is able to directly stimulate estradiol synthesis in ovarian granulosa cells (Janssens et al., 2000), and activation of GnRH receptors increases estradiol synthesis in the hippocampus, impacting dendritic spine density and levels of synaptic proteins (Fester et al., 2012; Prange-Kiel et al., 2013). Results of Experiment 3 in the present work, in which a GnRH-induced enhancement in hippocampus-dependent memory was blocked by inhibition of estradiol synthesis in the hippocampus, provide a functional implication for behavior of these neural effects.

In conclusion, results of the current study offer evidence to reconcile the conflicting data regarding the roles of circulating and hippocampus-derived estrogens on memory. They indicate that circulating estrogens impact memory through their ability to regulate hippocampal neuroestradiol synthesis. They also suggest that the negative impact of long-term ovarian hormone deprivation on memory is due to decreased levels of hippocampus-derived estrogens that would result from decreased levels of circulating estrogens. Future research should aim to further elucidate the relationship between circulating estradiol, the hypothalamus, and subsequent effects on the hippocampus, cognition, and other behavioral measures.

## Synthesis

Reviewing Editor: Margaret McCarthy, Univ of Maryland School of Medicine Decisions are customarily a result of the Reviewing Editor and the peer reviewers coming together and discussing their recommendations until a consensus is reached. When revisions are invited, a fact-based synthesis statement explaining their decision and outlining what is needed to prepare a revision will be listed below. Note: If this manuscript was transferred from JNeurosci and a decision was made to accept the manuscript without peer review, a brief statement to this effect will instead be what is listed below.

The authors have made a strong and cogent response to the previous reviews, which were generally positive. One unresolved issue is the question of what estradiol levels are in the hippocampus following treatment with the aromatase inhibitor, Letrozol. The authors note that they are trying to achieve this but do not trust the validity of the results they are getting. Indeed this is a problem that is plaguing the entire field right now as the only validated approach, the us of RIA, is not available anymore. The current Elisa kits available do not work for measuring steroids in brain and LS/MS is too insensitive. Therefore the authors are correct that this cannot be reliably achieved at this time.
